# Inhalable Nanoparticles/Microparticles of an AMPK and Nrf2 Activator for Targeted Pulmonary Drug Delivery as Dry Powder Inhalers

**DOI:** 10.1208/s12248-020-00531-3

**Published:** 2020-11-16

**Authors:** Maria F. Acosta, Michael D. Abrahamson, David Encinas-Basurto, Jeffrey R. Fineman, Stephen M. Black, Heidi M. Mansour

**Affiliations:** 1 Skaggs Pharmaceutical Sciences Center, The University of Arizona College of Pharmacy, 1703 E. Mabel St, Skaggs Pharmaceutical Sciences Center, Tucson, Arizona 85721-0207 USA; 2 Department of Pediatrics, University of California San Francisco School of Medicine, San Francisco, California USA; 3 University of California San Francisco Benioff Children’s Hospital, San Francisco, California USA; 4 University of California San Francisco Cardiovascular Research Institute, San Francisco, California USA; 5 Department of Medicine, Division of Translational and Regenerative Medicine, The University of Arizona College of Medicine, Tucson, Arizona USA; 6 Department of Medicine, Center for Lung Vascular Pathobiology, The University of Arizona College of Medicine, Tucson, Arizona USA; 7 Department of Physiology, The University of Arizona College of Medicine, Tucson, Arizona USA; 8 BIO5 Institute, The University of Arizona, Tucson, Arizona USA

**Keywords:** advanced spray drying, 2D/3D human lung cell cultures, *in vitro*, nanotechnology, respiratory drug delivery

## Abstract

Metformin is an activator of the AMPK and Nrf2 pathways which are important in the pathology of several complex pulmonary diseases with unmet medical needs. Organic solution advanced spray drying in the absence of water in closed-mode was used to design and develop respirable dry powders. Following comprehensive characterization, the influence of physicochemical properties was correlated with performance as aerosols using inertial impaction and three different human dry powder inhaler (DPI) devices varying in device properties. *In vitro* cell assays were conducted to test safety in 2D human pulmonary cell lines and in 3D small airway epithelia comprising primary cells at the air-liquid interface (ALI). In addition, *in vitro* transepithelial electrical resistance (TEER) was carried out. Metformin remained crystalline following advanced spray drying under these conditions. All SD powders consisted of nanoparticles/microparticles in the solid state. *In vitro* aerosol dispersion performance showed high aerosolization for all SD metformin powders with all DPI devices tested. High emitted dose for all powders with all three DPI devices was measured. Differences in other aerosol performance parameters and the interplay between the properties of different formulations produced at specific pump rates and the three different DPI devices were correlated with spray drying pump rate and device properties. Safety over a wide metformin dose range was also demonstrated *in vitro*. Aerosol delivery of metformin nanoparticles/microparticles has the potential to be a new “first-in-class” therapeutic for the treatment of a number of pulmonary diseases including pulmonary vascular diseases such as pulmonary hypertension.

**Supplementary Information:**

The online version contains supplementary material available at 10.1208/s12248-020-00531-3.

## INTRODUCTION

Metformin has long been used to treat type-2 diabetes mellitus and is a known AMP-activated protein kinase (AMPK) activator that is unrelated to its hypoglycemic actions (1). Since mitochondrial glucose oxidation is inhibited in patients with pulmonary hypertension (PH) and glucose levels are increased in endothelial and smooth muscle cells, metformin has been reported to have positive effects in the treatment of PH (2). Pulmonary hypertension affects adult and children (3). It is important to note that PH (4) is known to exist concomitantly with other pulmonary diseases including cystic fibrosis (CF) (5–7), IPF (8,9), and chronic obstructive pulmonary disease (COPD) (10).

In addition to being an AMPK activator, metformin is also a Nrf2 activator (11), increasing Nrf2 nuclear concentrations, affecting senescence, and delaying aging (11). Metformin has been shown to improve mitochondrial function by activating transcription of nuclear factor erythroid-related factor 2 (Nrf2) (12). Metformin has been reported to activate AMPK and Nrf2 signaling and induces the expression of antioxidant genes NQO1, γ-GCSm, HO-1, and SOD (13). Metformin alleviates Pb-induced mitochondrial fragmentation via antioxidative effects originating from AMPK/Nrf2 pathway activation promoting cell survival (14), prevents blood-brain barrier (BBB) endothelial cell dysfunction and loss of BBB integrity (15), and is neuroprotective (16,17). Nrf2 is known to have an important role in pulmonary diseases including acute lung injury (18,19) and idiopathic pulmonary fibrosis (IPF) (8). Thus, these important pharmacological properties make metformin a candidate to treat complex pulmonary diseases that continue to have unmet medical needs.

The pulmonary route (20,21) has long been the primary route used to treat pulmonary diseases as it offers several distinct advantages. These advantages are drug delivery in a targeted manner directly and non-invasively to the lungs with rapid onset of drug action locally and minimal systemic exposure leading to minimal systemic side effects, if any. In addition, a much lower drug dose is typically needed for inhalation aerosol delivery than for other routes due to a higher local concentration in the lungs. Moreover, drug action tends to be longer in the lungs by virtue of low drug metabolism, since the lung is inherently a low metabolic organ. Moreover, this route avoids first-pass metabolism in the liver and overcomes poor gastrointestinal (GI) absorption limitations. The different classes of inhaler devices used clinically to aerosolize drugs for targeted pulmonary delivery are dry powder inhalers (DPIs) (22–25), nebulizers (26,27), soft-mist inhalers (28–30), and pressurized metered-dose inhalers (pMDIs) (31–33).

DPI devices are smaller and easier to use than the other inhaler devices, have more versatility in device design, enable the formulation of poorly water-soluble drugs and/or drugs prone to rapid degradation as liquids, and can deliver multiple drugs simultaneously in the same aerosol as dual-drug DPIs and triple-drug DPIs which are currently FDA approved. DPIs are FDA approved for use in children as young as 4 years old. DPIs do not use propellants and there are chemical and physical stability advantages that the solid state provides (34,35). For inhalation powders (22,36), there are several important solid-state physicochemical particle properties (37) directly influencing aerosol dispersion as powders which are particle size, the surface morphology, the aerodynamic diameter, particle morphology, the degree of crystallinity, residual water content, and interparticulate interactions (38). The interparticulate interactions are electrostatic interactions, Van der Waals forces, mechanical interlocking, and capillary condensation all influence the aerosolization of powders. Since respiratory products are drug-device combination products, the formulation-device interactions impact performance. Hence, inhaler device properties directly influence aerosol properties. A variety of different types of DPI devices are available having different internal geometry, resistance, and shear stress properties (23,35).

The purpose of this study was to rationally design, develop, and optimize inhalable nanoparticles/microparticles of metformin for respiratory drug delivery as DPIs. Four spray drying pump rates of 25%, 50%, 75%, and 100% were used for solid-state particle engineering design by advanced organic solution spray drying in closed-mode and in the absence of water. Comprehensive solid-state physicochemical characterization was carried out. These advanced spray dried metformin powders were integrated with three different unit-dose capsule-based DPI devices varying in device geometry, resistance, and shear stress properties and that are FDA approved for human use. *In vitro* aerosol dispersion properties were measured using inertial impaction to quantify aerodynamic size and aerodynamic size distribution for predictive lung deposition modeling into specific lung regions. *In vitro* cell viability was measured as a function of dose using 2D human pulmonary cell lines from different lung regions. In addition, *in vitro* cell viability assays were performed using human primary cells cultured as 3D small airway epithelia at an air-liquid interface (ALI). *In vitro* transepithelial electrical resistance (TEER) in air-interface culture (AIC) conditions was also conducted. To the authors’ knowledge, this is the first to report the generation of SD metformin particles that could be utilized to treat complex pulmonary disease.

## MATERIALS AND METHODS

### Materials

Metformin hydrochloride (Metformin) [C_4_H_111_N_5._HCl] is a small molecular weight (MW) drug with a MW of 165.625 g/mol g/mol and was purchased from Spectrum Chemical MFG CORP (New Brunswick, NJ, USA). Its chemical structure is shown in Supplementary Material Fig. 1 (ChemDraw™ Ultra Ver. 15.0.; CambridgeSoft, Cambridge, MA, USA). Methanol (HPLC grade, ACS-certified grade, purity 99.9%) and chloroform (HPLC grade, ACS-certified grade, purity 99.8%) were obtained from Fisher Scientific (Fair Lawn, NJ, USA). Hydranal®-Coulomat AD and resazurin sodium salt were from Sigma-Aldrich (St. Louis, MO, USA). Raw metformin powder was stored in a sealed glass desiccator over indicating Drierite/Drierite™ desiccant under ambient pressure. Other chemicals were stored also under room conditions. Ultra-high purity (UHP) nitrogen gas was used for all experiments and it was obtained from The University of Arizona Cryogenics and Gas facility (Tucson, AZ, USA).

Human pulmonary cell lines were purchased from the American Type Culture Collection ATCC® (Manassas, VA, USA). These human pulmonary cell lines are A549 (ATCC® CCL-185™), NCI-H358 (ATCC® CRL-5807™), and Calu-3 (ATCC® HTB-55™). The Eagle’s minimum essential medium (EMEM) was also purchased from ATCC® (Manassas, VA, USA). Dulbecco’s modified Eagle’s medium (DMEM), Advanced 1X, fetal bovine serum (FBS), Pen-Strep, Fungizone®, and l-glutamine were obtained from Gibco® by Life Technologies (Thermo Fisher Scientific Inc., Waltham, MA, USA). Small Air™ is a unique 3D human small airway epithelium reconstituted *in vitro* and its SmallAir™ special growth media (which is serum free and contains growth factors and phenol red) were both purchased from Epithelix (Geneva, Switzerland).

### Methods

#### Preparation of Respirable Powders by Organic Solution Advanced Spray Drying (No Water) in Closed-Mode

Organic solution advanced spray drying (SD) in the absence of water, as previously reported (39,40), was utilized to develop dry particles of metformin. Specifically, a Büchi B-290 Mini Spray Dryer from Büchi Corporation (Büchi Labortechnik AG, Flawil, Switzerland) with a high-performance cyclone in closed mode using UHP dry nitrogen gas as the atomizing and drying gas and connected to a B-295 Inert Loop (Büchi Labortechnik AG, Flawil, Switzerland) was employed. The SD conditions were tailored in order to get the most optimum particles. Metformin 0.1% w/v in methanol solutions was spray dried at four rationally chosen spray drying pump rates (PR) of 25% (low), 50% (medium), 75% (medium-high), and 100% (high). The drying gas atomization rate was 670 L/h at 35 mmHg, aspiration rate of 35 m^3^/h at 100% rate, and an inlet temperature of 150°C. The diameter of the stainless-steel nozzle was 0.7 mm. All these parameters were maintained constant during all the experiments. Supplementary Material Table 1 lists the spray drying conditions and Supplementary Material Table 2 lists the corresponding outlet temperatures. The SD particles were separated from the nitrogen drying gas in the high-performance cyclone and collected in a small sample collector. All SD powders were carefully stored in sealed scintillation glass vials and stored in sealed desiccators over indicating Drierite/Drierite™ desiccant at − 20°C.

#### Laser Light Diffraction Particle Sizing and Size Distribution

As previously reported (39,40), the mean particle size and distribution were determined by ultraviolet (UV) laser diffraction using the SALD-7101 (Shimadzu, Japan) nanosizer. SD metformin particles were dispersed in chloroform and sonicated for 5 s before the analysis in order to break up the agglomerates. A quartz glass cell was used under stirred conditions. The low refractive index 1.35–0.10 was used. Number-based dimension of particle amount distribution was obtained for samples. In addition to acquiring the particle size distributions, the *D*_v10_, *D*_v50_, and *D*_v90_ parameters were measured. The span value was calculated using Eq. 1 defined as:

1 $$\mathrm{Span}=\left[\left({D}_{\mathrm{v}90}-{D}_{\mathrm{v}10}\right)/{D}_{\mathrm{v}50}\right]$$

#### Scanning Electron Microscopy

The visual imaging, the analysis of particle morphology, particle size, surface morphology, and other microscopic characteristics were achieved by scanning electron microscopy (SEM) using a FEI Inspect S microscope (FEI, Brno, Czech Republic). The conditions have been previously reported (41). Samples were placed on double-sided adhesive carbon tabs (TedPella, Inc. Redding, CA, USA), which were adhered to aluminum stubs (TedPella, Inc.) and were coated with a gold thin film using a Hummer 6.2 sputtering system from Anatech (Union City, CA, USA). The coating process was operated at 15 AC milliAmperes with about 7 kV of voltage for 90 s. The electron beam with an accelerating voltage of 30 kV was used at a working distance of ~ 9–12 mm. Several magnification levels were used.

#### Particle Sizing and Size Distribution by SEM Image Analyses

In order to compare the mean size and standard deviation from imaging with the particle size obtained from the laser light diffraction particle sizes, representative micrographs for each SD powder at × 5000 magnification were analyzed by measuring the diameter of at least 100 particles per sample using SigmaScan™ Pro 5.0.0 (Systat, Inc., San Jose, CA, USA), as previously reported (41–44).

#### X-Ray Powder Diffraction

Powder crystallinity was determined by X-ray powder diffraction (XRPD). Using similar conditions as previously reported (41), XRPD patterns of raw metformin and SD metformin powders were collected at room temperature with a PANalytical X’pert diffractometer (PANalytical Inc., Westborough, MA, USA) equipped with a programmable incident beam slit and an X’Celerator Detector. The X-ray radiation used was Ni-filtered Cu Kα (45 kV, 40 Ma, and *λ* = 1.5418 Å). Measurements were taken between 5.0 and 50.0° (2θ) with a scan rate of 2°/min. The powder samples were loaded on zero background silicon sample holder.

#### Differential Scanning Calorimetry

A TA Q1000 differential scanning calorimeter (DSC) (TA Instruments, New Castle, DE, USA) equipped with T-Zero® technology, RSC90 automated cooling system, and an autosampler, using similar conditions as previously reported (41), was used to perform thermal analysis and phase transition measurements for the metformin samples. The instrument was previously calibrated with indium. Approximately 1–3 mg of powder was weighed and placed into anodized aluminum hermetic DSC pans. The T-Zero® DSC pans were hermetically sealed with the T-Zero® hermetic press (TA Instruments, New Castle, DE, USA). For all the experiments, an empty hermetically sealed aluminum pan was used as reference. UHP nitrogen gas was used as the purging gas at a rate of 40 mL/min. The samples were heated from 0.00 to 250.00°C at a scanning rate of 5.00°C/min. All measurements were carried out in triplicate (*n* = 3).

#### Hot-Stage Microscopy Under Cross-Polarizers

As described previously (41), hot-stage microscopy (HSM) was performed using a Leica DMLP cross-polarized microscope (Wetzlar, Germany) equipped with a Mettler FP 80 central processor heating unit and Mettler FP82 hot stage (Columbus, OH, USA). Samples were fixed on a glass slide and heated from at 25.0 to 250.0°C at a heating rate of 5.00°C/min. The images were digitally captured using a Nikon Coolpix 8800 digital camera (Nikon, Tokyo, Japan) under × 10 optical objective and × 10 digital zoom.

#### Karl Fischer Titration

Using conditions similar to those previously reported (41), the residual water content of SD powders was chemically quantified by coulometric Karl Fischer titration (KFT) using a TitroLine 7500 trace titrator (SI Analytics, Weilheim, Germany). Approximately 1–5 mg of powder was added to the titration cell containing Hydranal® Coulomat AD reagent.

#### Raman Spectroscopy

Using similar conditions previously reported (41), Raman (45) spectra were obtained at 514-nm laser excitation using Renishaw InVia Reflex (Gloucestershire, UK) at the surface using a × 20 magnification objective on a Leica DM2700 optical microscope (Wetzlar, Germany) and equipped with a Renishaw inVia Raman system (Gloucestershire, UK). This Renishaw system had a 2400 l/mm grating, with a slit width of 65 μm and a thermoelectrically cooled Master Renishaw CCD detector. The laser power was adjusted to achieve 5000 counts per second for the 520 cm^−1^ line of the internal Si reference. Raman spectra were acquired with 1% of laser power and 10 s of exposure for all samples.

#### Attenuated Total Reflectance-Fourier Transform Infrared Spectroscopy

A Nicolet Avatar 360 FTIR spectrometer (Varian Inc., CA, USA) equipped with a DTGS detector and a Harrick MNP-Pro (Pleasantville, New York, USA) attenuated total reflectance (ATR) accessory was used for this kind of spectroscopy. Each spectrum was collected for 32 scans at a spectral resolution of 2 cm^−1^ over the wavenumber range of 4000–400 cm^−1^. A background spectrum was carried out under the same experimental conditions. Spectral data were acquired with EZ-OMNIC software. These conditions were similar to those previously reported (41).

#### *In vitro* Dry Powder Inhaler Aerosol Dispersion Performance

The aerosol dispersion performance of SD metformin formulations was tested using the Next Generation Impactor™ (NGI™) (MSP Corporation, Shoreview, MN, USA) with a stainless steel induction port (USP throat) attachment (NGI Model 170; MSP Corporation) equipped with specialized stainless steel NGI gravimetric insert cups (MSP Corporation), according to USP Chapter <601> specifications on aerosols (46). Three different FDA-approved human DPI devices: (a) HandiHaler® (Boehringer Ingelheim, Ingelheim, Germany), (b) NeoHaler™ (Novartis AG, Stein, Switzerland), and (c) Aerolizer® (Novartis Pharma AG, Basle, Switzerland) were tested. Using similar conditions as reported previously (41), the experiments were conducted with an airflow rate (*Q*) of 60 L/min, which was adjusted and measured before each experiment using a Copley DFM 2000 digital flow meter (Copley Scientific, Nottingham, UK). The NGI™ was connected to a Copley HCP5 high-capacity vacuum pump through a Copley TPK 2000 critical flow controller (Copley Scientific, Nottingham, UK). The mass of powder deposited on each stage was gravimetrically quantified using type A/E glass fiber filters with diameter 55 mm (PALL Corporation, Port Washington, New York, USA) and 75 mm (Advantec, Japan). Quali-V clear HPMC size 3 inhalation grade capsules (Qualicaps, NC, USA) were filled with ~ 10 mg of powder. Three capsules were used in each experiment. *In vitro* aerosolization was conducted in triplicate (*n* = 3) under ambient conditions.

At *Q* = 60 L/min, the D_a50_ aerodynamic cutoff diametforminer for each NGI stage was calibrated by the manufacturer and stated as follows: stage 1 (8.06 μm), stage 2 (4.46 μm), stage 3 (2.82 μm), stage 4 (1.66 μm), stage 5 (0.94 μm), stage 6 (0.55 μm), and stage 7 (0.34 μm). The emitted dose (ED) was determined as the difference between the initial mass of powder loaded in the capsules and the remaining mass of powder in the capsules following the aerosolization. The emitted dose, ED (%), was used to express the percentage of ED based on the total dose (TD) used (Eq. 2). The fine particle dose (FPD) was defined as the dose deposited on stages 2 to 7. The fine particle fraction, FPF (%), was expressed as the percentage of FPD to ED (Eq. 3). The respirable fraction, RF (%), was used as the percentage of FPD to total deposited dose (DD) on all impactor stages (Eq. 4).

2 $$\mathrm{Emitted}\ \mathrm{dose}\ \mathrm{fraction}\ \left(\mathrm{ED}\%\right)=\frac{\mathrm{ED}}{\mathrm{TD}}\times 100\%\kern0.5em$$

3 $$\mathrm{Fine}\ \mathrm{particle}\ \mathrm{fraction}\ \left(\mathrm{FPF}\%\right)=\frac{\mathrm{FPD}}{\mathrm{ED}}\times 100\%$$

4 $$\mathrm{Respirable}\ \mathrm{fraction}\ \left(\mathrm{RF}\%\right)=\frac{\mathrm{FPD}}{\mathrm{DD}}\times 100\%$$

In addition, the mass median aerodynamic diameter (MMAD) of aerosol particles and geometric standard deviation (GSD) was calculated using a Mathematica (Wolfram Research, Inc., Champaign, IL, USA) program written by Dr. Warren Finlay.

#### *In vitro* Cell Dose Response Assay in a 2D Cell Culture

The effects of SD metformin formulations on the viability of human representative pulmonary cell lines exposed to different concentrations were tested, using similar conditions previously reported (42,47). A549 (a human alveolar epithelial lung adenocarcinoma cell line) and H358 (a bronchioalveolar carcinoma pulmonary cell line) were used as models of the alveolar type I alveolar epithelial cells and alveolar type II cells which express lung surfactant-associated protein A (SP-A), respectively (48). These cell lines were grown in a growth medium including Dulbecco’s modified Eagle’s medium (DMEM), Advanced 1x, 10% (v/v) fetal bovine serum (FBS), Pen-Strep (100 U/mL penicillin, 100 μg/mL), Fungizone (0.5 μg/mL amphotericin B, 0.41 μg/mL sodium deoxycholate), and 2 mM l-glutamine in a humidified incubator at 37°C and 5% CO_2_.

After confluence, A549 and H358 cells were seeded in 96-black well plates at a concentration of 5000 cells/well and 100 μL/well. They were incubated for 48 h to allow attachment to the surface of the plates. Cells were then exposed to different concentrations of the raw and SD formulations. The drug solutions were prepared by dissolving the powders in DMEM media. A volume of 100 μL of the different drug solution concentrations was added to each well. Seventy-two (72) hours after exposure under incubation at 37°C and 5% CO_2_, 20 μL of 20 μM resazurin sodium salt were added to each well and incubated for 4 h. At this point, the fluorescence intensity of resorufin, which can only be produced by viable cells from resazurin, was detected at 544 nm (excitation) and 590 nm (emission), using the Synergy H1 Multi-Mode Reader (BioTek Instruments, Inc., Winooski, VT, USA). The relative viability of the cells was calculated as follows by Eq. 5:

5 $$\mathrm{Relative}\ \mathrm{viability}\ \left(\%\right)=\frac{\mathrm{Sample}\ \mathrm{fluorescence}\ \mathrm{intensity}}{\mathrm{Control}\ \mathrm{fluorescence}\ \mathrm{intensity}}\times 100\%$$

Analysis of variance (ANOVA) statistical method was used to compare the relative viability between the treated vs. the non-treated cells using SigmaPlot 13 (SYSTAT Software, Inc., San Jose, CA) scientific software.

#### *In vitro* Transepithelial Electrical Resistance Analysis upon Particle Exposure to Lung Epithelial Cells

Calu-3 cells were grown in a growth medium including Eagle’s minimum essential medium (EMEM), 10% (v/v) fetal bovine serum (FBS), Pen-Strep (100 U/mL penicillin, 100 μg/mL), and Fungizone (0.5 μg/mL amphotericin B, 0.41 μg/mL sodium deoxycholate) in a humidified incubator at 37°C and 5% CO_2_, using previously reported similar conditions (42,47) After confluence, the cells were seeded at a concentration of 500,000 cells/mL in Costar Transwells inserts® (0.4-μm polyester membrane, 12 mm for a 12-well plate) from Fisher Scientific (Hampton, NH, USA) with 0.5 mL of media on the apical side and 1.5 mL of media on the basolateral side. After the TEER values reached 500 Ω cm^2^, indicating a confluent monolayer at the ALI, the cells were exposed to 1000 μM of representative SD formulations dissolved in non-supplemented EMEM media. The liquid aerosol formulations were delivered to the Calu-3 cells at the ALI using a Penn-Century MicroSprayer® Aerosolizer Model IA-1B (Philadelphia, PA, USA) (42,48). TEER values were then recorded after 3 h of exposure and then every 24 h up to 7 days after drug exposure, as previously reported (42,47) using an EndOhm 12 mm Culture Cup (World Precision Instruments, Sarasota, FL, USA).

#### *In vitro* Cell Dose Response Assay in a 3D Cell Culture at the Air-Liquid Interface

The 3D small airway human epithelia, SmallAir™, comprise human primary cells that were fully differentiated and functional. The cells were received in 24-well Transwell inserts® from Epithelix (Geneva, Switzerland) in a gel matrix. Once the fully differentiated cells were received, they were transferred into a new 24-well plate with 700 μL of the SmallAir™ media in the basal surface to create the ALI. Media was changed every other day.

Experiments were performed after 3 days of incubation at 37°C and 5% CO_2_. For *in vitro* cell dose response, cells were exposed to a 1000 μM solution of SD metformin (25% PR). After 72 h of incubation, the inserts were rinsed with a 6 μM resazurin solution in order to eliminate the remaining red phenol from the cell growth media. The inserts were transferred to a new 24-well plate filled with 500 μL/well of resazurin solution. A volume of 200 μL per each well was added in the apical surface. After 1 h of incubation, 100 μL from the apical side was transferred to a 96-black well plate. At this point, the fluorescence intensity of resorufin was detected at 544 nm (excitation wavelength) and 590 nm (emission wavelength) using the Synergy H1 Multi-Mode Reader (BioTek Instruments, Inc., Winooski, VT, USA). The relative viability of cell line was calculated with Eq. 5. This protocol was provided by the vendor (49).

#### *In vitro* Transepithelial Electrical Resistance Analysis upon Particle Exposure to 3D Human Small Airway Epithelia at the Air-Liquid Interface

After receiving SmallAir™ 3D airway epithelia comprising fully differentiated human primary cells and following the vendor’s protocol (49), they were transferred to 24-well plates pre-filled with 700 μL of SmallAir™ media in the basal side to create the ALI. After 3 days of incubation, 1000 μM solution of SD metformin (25% PR) was added to the cells. TEER values were measured using EVOMX (Epithelial VoltOhmMeter) and electrode (STX2) (World Precision Instruments, Sarasota, FL). To measure TEER, 200 μL of the cell media were added to the apical surface of the inserts. The long part of the electrode was inserted through the gap of the insert and leaned on the bottom of the well, and the short stem was above in the apical surface, inside the culture media. TEER values were obtained before exposure to the drug solution and after exposure to them. The response was measured after 3 h of exposure and then every 24 h for 5 days. Every time, the TEER measurement was finished, the media was removed from the apical surface in order to leave the cells in ALI conditions.

#### Statistical Analysis

Design of experiments (DoEs) was conducted using Design-Expert® 8.0.7.1 software (Stat-Ease Corporation, Minneapolis, MN, USA). A multi-factorial design for SD metformin was utilized for *in vitro* aerosol testing. Interaction of the inhaler device resistance and spray drying PR were evaluated using the analysis of variance (ANOVA) test performed using Design-Expert® software. The different interactions on the performance of the formulations were evaluated using the 3D surface plot generated from Design-Expert® 8.0.7.1 software. All experiments were performed in at least triplicate (*n* = 3) unless otherwise stated such as the *in vitro* cell viability (*n* = 6). Results were expressed as mean ± standard deviation. ANOVA statistics was used to compare the relative cell viability between the treated vs. the non-treated cells using SigmaPlot 13 (SYSTAT Software, Inc) scientific software.

## RESULTS

### Preparation of Respirable Powders by Organic Solution Advanced Spray Drying (No Water) in Closed-Mode

Particles were successfully produced under the advanced spray drying conditions described in the “Methods” section at 25%, 50%, 75%, and 100% PR.

### Laser Light Diffraction Particle Sizing and Size Distribution

The particle size data and the calculated span are summarized in Supplementary Material Table 3. All formulations showed narrow and unimodal particle size distributions. This was reflected in the calculated span values which were small and reflected of unimodal particle size distribution. All mean sizes were in the nanometer size range. The span values were similar for all formulations.

### Scanning Electron Microscopy

**Fig. 1:**
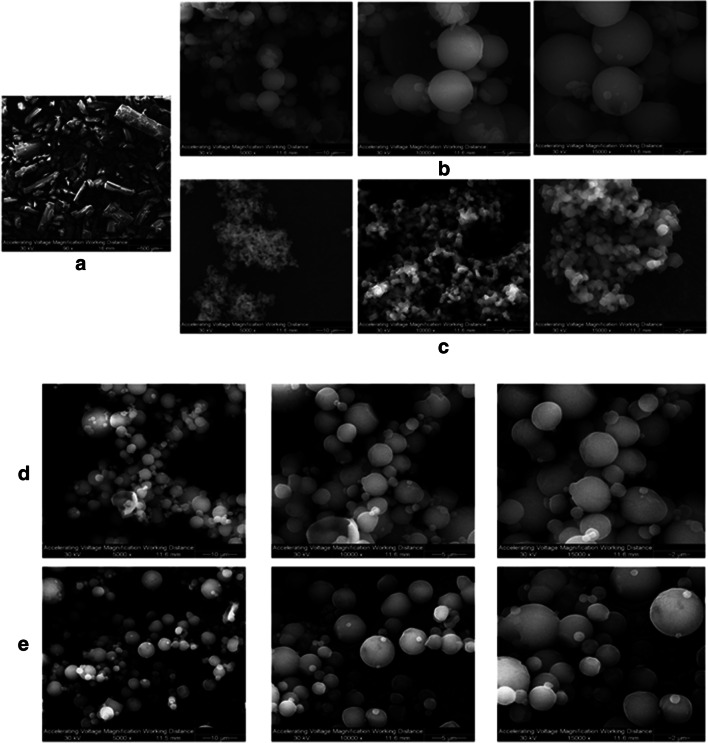
SEM micrographs of **a** raw metformin HCl, **b** SD metformin (25% PR), **c** SD metformin (50% PR), **d** SD metformin (75% PR), and **e** SD metformin (100% PR)

The SEM micrographs showed a tremendous change in particle shape and size between the raw metformin and the SD formulations. Raw metformin had needle shape (Fig. 1a) and the particle size was about 500 μm and beyond. The SD powders displayed spherical shapes in all pump rates. The smooth surface was more visible at 25%, 75%, and 100% PR (Fig. 1b–e).

### Particle Sizing and Size Distribution Using SEM Micrographs

The size range was from the nanometer to the low micrometer size for all SD systems. From Supplementary Material Table 4, it can be noted that the particles with the smallest mean diameter were the ones formulated at 50% PR and the 25% PR powders had the largest mean diameter. The widest range was for the SD metformin at 25%, whereas the narrowest was for the SD metformin at 50% PR.

### X-Ray Powder Diffraction

**Fig. 2:**
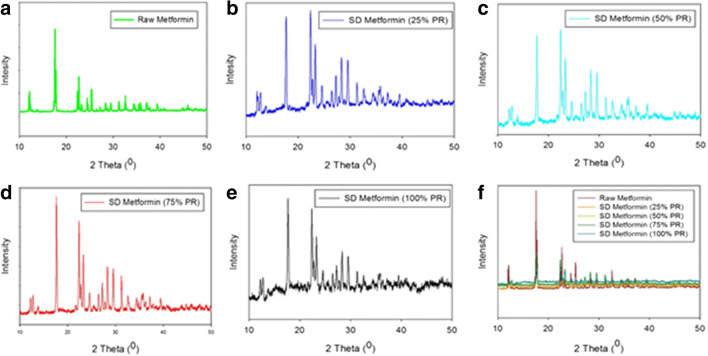
XRPD diffraction patterns of **a** raw metformin HCl, **b** SD metformin (25% PR), **c** SD metformin (50% PR), **d** SD metformin (75% PR), **e** SD metformin (100% PR), and **f** all

The XRPD diffraction patterns of raw and SD metformin presented sharp and intense peaks, characteristic of the long-range molecular order. As presented in Fig. 2, all raw and SD formulations had the same diffraction pattern, and the sharp, intense, and most notably representative peaks at 2θ angles were 17°, 22°, 23°, 31°, and 45°. This is in agreement with previous reports (50,51).

### Differential Scanning Calorimetry

**Fig. 3:**
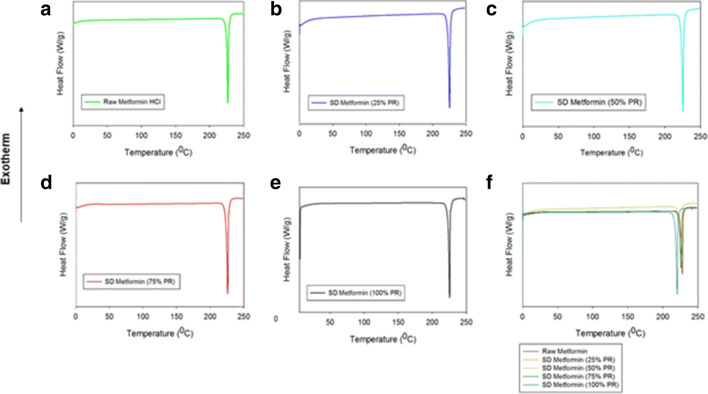
DSC thermograms of **a **raw metformin HCl, **b **SD metformin (25% PR), **c **SD metformin (50% PR), **d **SD metformin (75% PR), **e **SD metformin (100% PR), and **f **all

All raw and SD formulations had very similar thermograms (Fig. 3) at a scan rate of 5.00°C/min. There was only one main phase transition (i.e., a molecular order-to-disorder phase transition) at ~ 225°C, which corresponded to the melting of the drug (50,51) from the solid state to the liquid state. The enthalpy and temperature values are summarized in Supplementary Material Table 5.

### Hot Stage Microscopy Under Cross-Polarizers

**Fig. 4:**
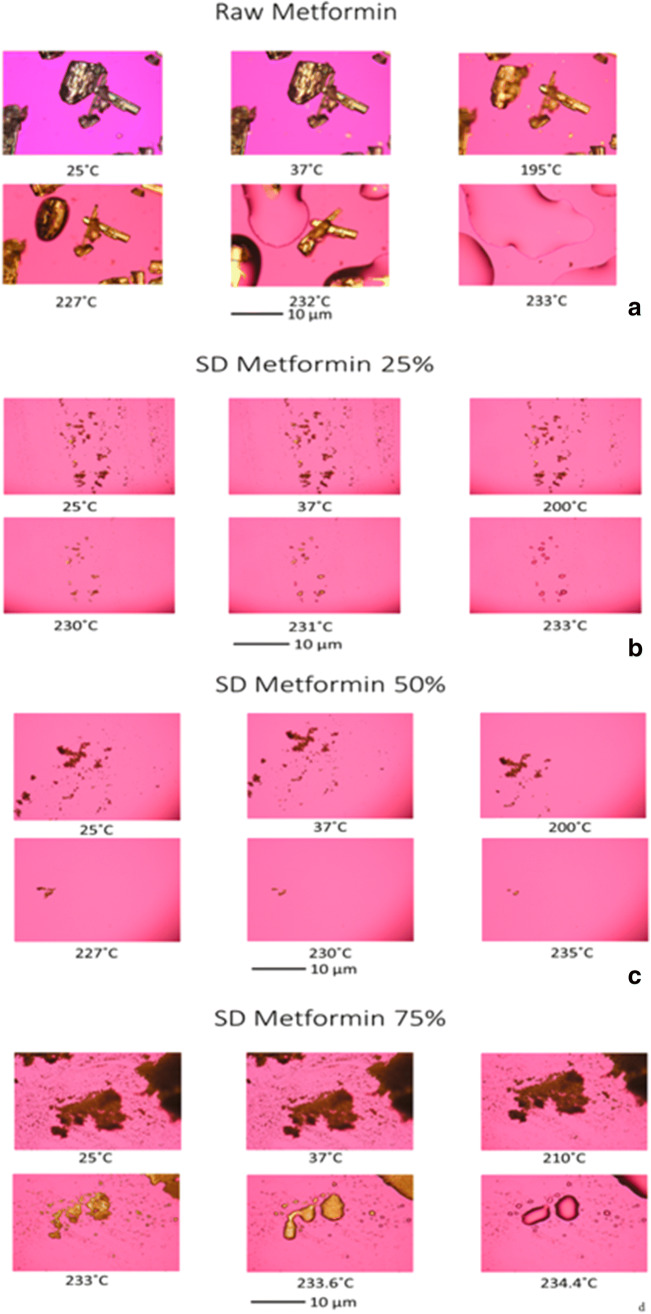
Representative HSM images of **a **raw metformin HCl and **b **SD metformin (25% PR). Scale bar = 10 μm

The images from the HSM were in agreement with the DSC and the XRPD results (Fig. 4). Birefringence was clearly present in all samples when observed under the microscope. There was only one phase transition seen and it was at a temperature ~ 226°C. The disappearance of the birefringence and the formation of the droplets were visual confirmation of drug melting.

### Karl Fischer Titration

The residual water content in all systems was quantified by Karl Fischer coulometric titration. In general, the values were low for all systems and ranged from 1.31–3.41%, as listed in Supplementary Material Table 6.

### Attenuated Total Reflectance-Fourier Transform Infrared Spectroscopy

The raw and SD powders produced nearly identical spectra (Supplementary Material Fig. 2). The prominent and typical bands were at 3369, 3294, 3155, 1626, and 1567 cm^−1^. These spectra are in agreement with previous reports (50,51).

### Raman Spectroscopy

The Raman spectra for all raw and SD formulation were the same (Supplementary Material Fig. 3). Characteristic Raman bands appeared in different wavelengths of the spectrum. The most representative for metformin were at 3375, 3197, 3301, 2821, 1472, 1169, 1087, 1043, 912, and 634 cm^−1^. These Raman spectra are in agreement with previous reports (50,51).

### *In vitro* Aerosol Dispersion Performance

**Fig. 5:**
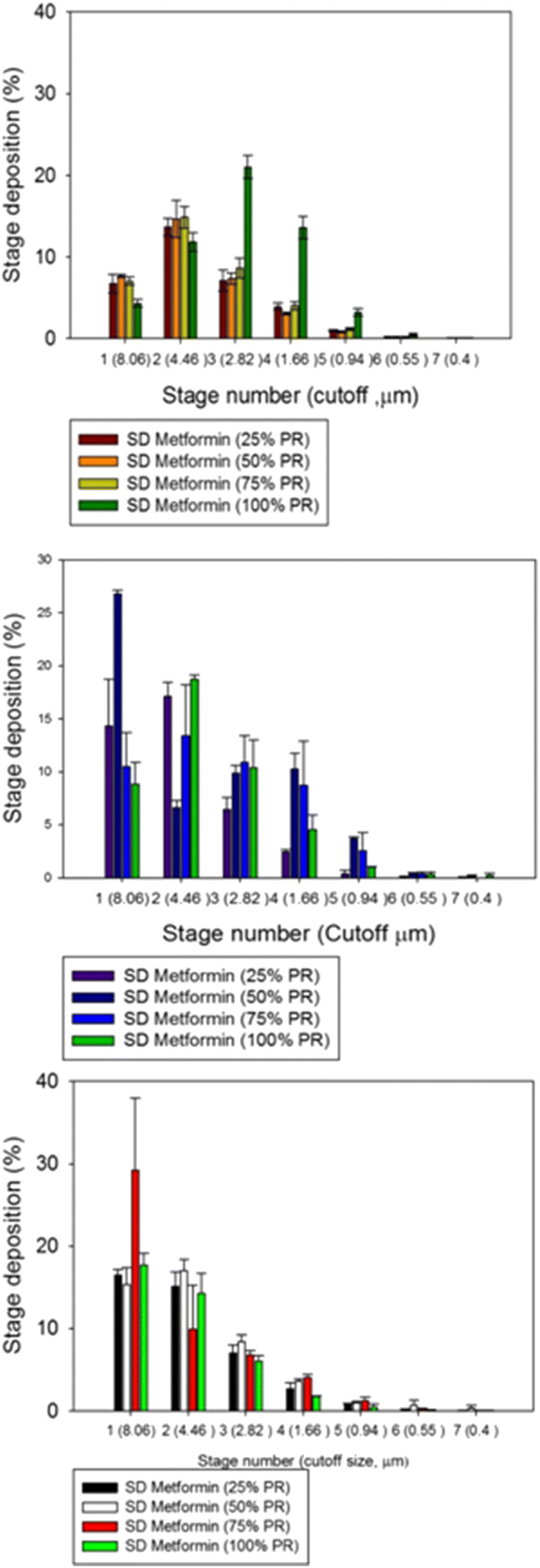
*In vitro* aerosol dispersion performance for various SD Met powders with three different human DPI devices: **a **Aerolizer®, **b **NeoHaler™, and **c ** HandiHaler®. (*n* = 3, mean ± SD)

The aerosol dispersion performance of the SD powders was tested following the USP chapter <601> specifications on aerosols as described above. Three different FDA-approved human DPI devices with different internal geometry, resistance, and shear device properties were used for this test. Aerosol deposition on each NGI™ stage was measurable, and deposition on the lower stages of stage 2 all the way to STAGE 7 (the lowest stage) was observed (Fig. 5). As listed in Supplementary Material Table 7, the ED values of all formulations remained very high for all formulations with the three devices. The ED values were close to 99% for all systems taking into consideration the standard deviations. The FPF values were also very similar for all formulations with the three devices. The FPF values ranged between ~ 25 and 35%, except for SD metformin at 100% PR using the Aerolizer® human DPI device which showed an FPF value of 52%. Regarding the RF values, there were variations between DPI devices. Using the HandiHaler®, a high shear stress human DPI device, and the NeoHaler™, a medium shear stress human DPI device, the RF values were lower than using the Aerolizer®, a low-medium shear stress device. The values ranged between 40–66% and 77–92%, respectively. The MMAD and GSD values were very similar in all formulations and among the three different devices. However, the MMAD calculated values as a result of using the HandiHaler® device were a bit larger (~ 7 μm) than from the Aerolizer® and the NeoHaler™ (~ 5 μm).

### *In vitro* Cell Dose Response Assay in a 2D Cell Culture

**Fig. 6:**
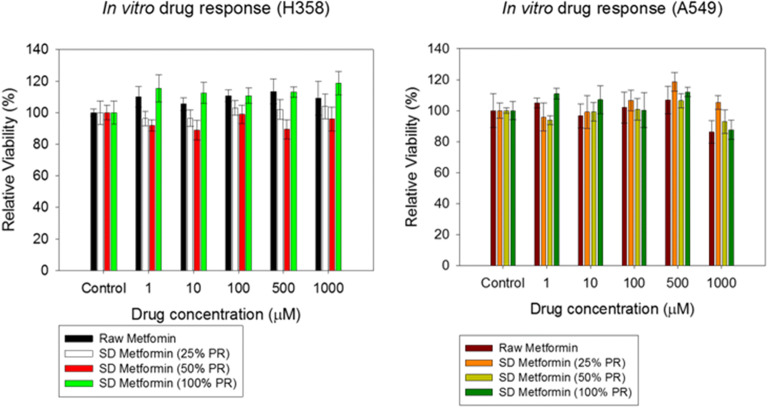
*In vitro* cell viability on human pulmonary cell lines **a **H358 and **b **A549, and cells after 72 h of exposure to different concentrations of raw metformin HCl and SD metformin. (*n* = 6, mean ± SD)

The plots shown in Fig. 6 show that there was no decrease in the viability in human pulmonary cell lines from either the human bronchioalveolar-(H359) or alveolar-(A549) lung regions 72 h after exposure to increasing concentrations of the raw and SD metformin. Indeed, the two cell lines remained viable at all concentrations.

### *In vitro* Transepithelial Electrical Resistance Analysis upon Particle Exposure to Lung Epithelial Cells

**Fig. 7:**
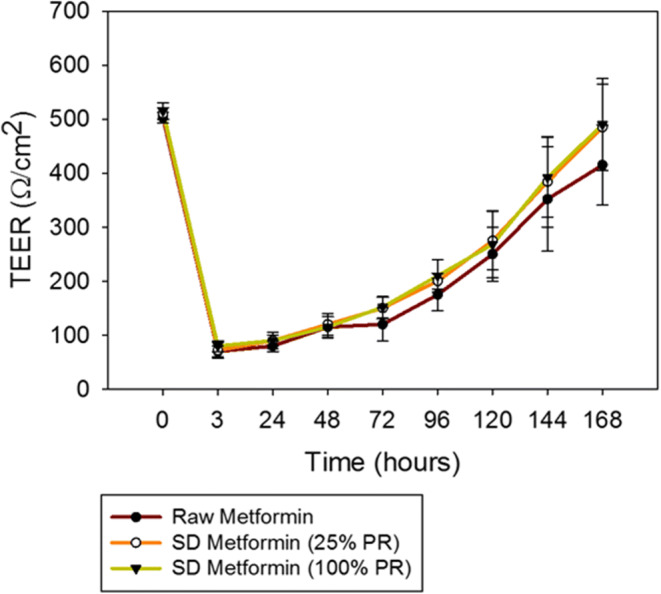
*In vitro* transepithelial electrical resistance (TEER) analysis of Calu-3 human lung bronchial epithelial cell line at the air-liquid interface (ALI) exposed to 1,000 micromolar concentration of raw metformin HCl and SD metformin using a Penn-Century MicroSprayer® Aerosolizer Model IA-1B (*n* = 3, mean ± SD)

After the exposure of the cells to the formulations, the integrity of the Calu-3 bronchial large airway cellular monolayer in AIC conditions was disrupted, as seen in Fig. 7. This was reflected in the decrease of the TEER values. However, over time, the cell monolayer recovered to pre-exposure values.

### *In vitro* Cell Dose Response Assay in a 3D Primary Cell Culture

**Fig. 8:**
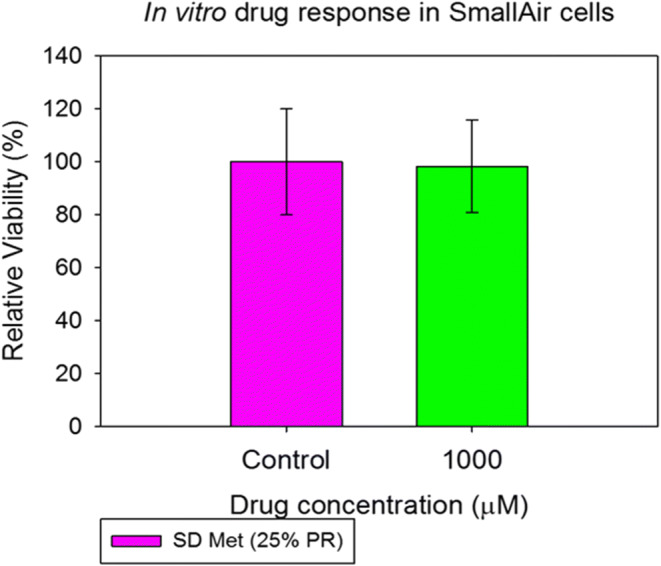
*In vitro* cell viability for SmallAir™ 3D human pulmonary primary cells at the air-liquid interface (ALI) after 72 h of exposure to SD metformin (25% PR). (*n* = 3, mean ± SD)

After 72 h of exposure of the SmallAir™ 3D human pulmonary primary cells to the solution of SD metformin, they remained viable indicating no adverse effects at the delivered dose (Fig. 8).

### *In vitro* Transepithelial Electrical Resistance Analysis upon Particle Exposure to 3D Human Small Airway Epithelia

**Fig. 9:**
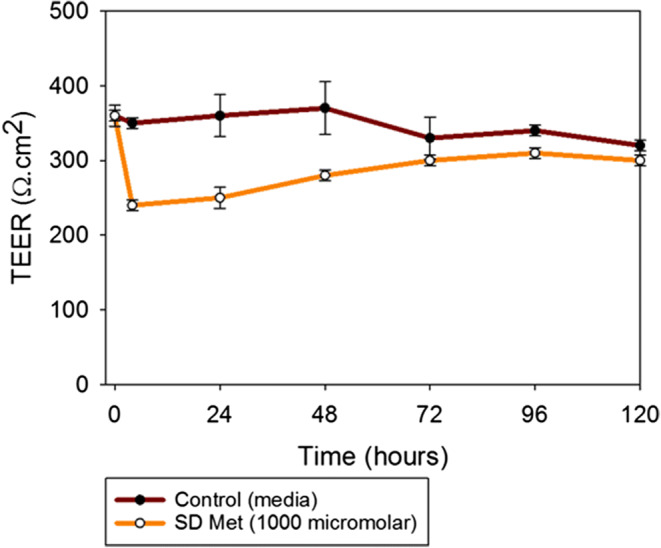
*In vitro* transepithelial electrical resistance (TEER) analysis of SmallAir™ human pulmonary primary cells at the air-liquid interface (ALI) exposed to 1,000 micromolar concentration of SD metformin at the ALI using a micropipette. (*n* = 3, mean ± SD)

As seen in Fig. 9, the TEER values of the SmallAir™ 3D human pulmonary primary cells after the exposure to SD metformin did not decrease below 200 Ω cm^2^.

## DISCUSSION

To the authors’ knowledge, this is the first study to report these findings. Organic solution advanced spray drying from dilute solution using rationally selected spray drying conditions allowed us to successfully develop dry powders of metformin as nanoparticles and microparticles in the solid state. It has been previously demonstrated that solid-state formulations used in DPIs must have essential physicochemical properties to achieve proper aerosol dispersion as powders to allow targeted lung delivery and deposition. Interfacial and interparticulate interactions strongly influence powder flow and aerosol dispersion (35). By tailoring the essential particle properties, interparticulate interactions can be reduced to produce high aerosol dispersion performance. Specifically, SD metformin particle morphology was shown to be spherical and the surface morphology was found to be smooth, reducing interparticulate interactions, avoiding the formation of agglomerates due to mechanical interlocking and/or electrostatic forces. From the SEM micrographs (Fig. 2), it can be seen that particles formed at 75% PR and 100% PR were more spherical, had a smoother surface, and presented less degree of agglomeration than the SD metformin 25% PR and SD metformin 50% PR powders. This correlates the pump rate with particle properties in a meaningful manner.

Moreover, minimizing residual water content prevents capillary condensation between particles and therefore prevents agglomeration. Supplementary Material Table 6 shows that all powders had low residual water content and were well accepted for dry powders intended for inhalation. It is noted that relatively higher residual water content was present for powders produced at the low spray drying pump rate. Perhaps, this might be possibly due to differences in surface area which would lend to having more binding sites on the surface for water vapor adsorption to occur. All SD formulations had the essential solid-state particle properties required for inhalable powders. Specifically, these solid-state particles had very low residual water content, spherical particle morphology, smooth surface morphology, and particle sizes in the respirable size range as inhalable nanoparticles/microparticles.

The XRPD diffraction patterns (Fig. 2) showed intense and sharp peaks indicative of long-range molecular order in the raw metformin and all SD metformin powders. A polymorph interconversion may have occurred to enable the formation of the crystalline state following advanced spray drying under these conditions. Crystallinity was further confirmed by DSC (Fig. 3 and Supplementary Material Table 5). A single main order-to-disorder phase transition of melting from the solid to liquid was clearly observed. Birefringence, a visual characteristic of crystals, was observed in HSM and further confirmed retention of crystallinity following advanced spray drying under these conditions. The absence of glass transition temperature T_g_, HSM birefringence visualization, and XRPD peaks all indicated that the SD powders remained crystalline after the spray drying process under these conditions.

The ATR-FTIR (Supplementary Material Fig. 2) and Raman (Supplementary Material Fig. 3) spectra were identical before and after spray drying under the reported conditions; hence, there was no change in the solid-state vibrational bonding structures following spray drying under these conditions. From ATR-FTIR (Supplementary Material Fig. 2), the characteristic bands observed at 3369 cm^−1^ and 3294 cm^−1^ corresponded to the N–H primary stretching vibration and the band at 3155 cm^−1^ was due to the N– secondary stretching; the characteristic bands at 1626 cm^−1^ and 1567 cm^−1^ belonged to C–N stretching (50,51). The Raman spectra (Supplementary Material Fig. 3) also showed many characteristic bands that complemented the ATR-FTIR spectra (Supplementary Material Fig. 2). In this case, the spectrum before and after SD was identical also. The bands encountered at ~ 3375, 3197, and 3301 cm^−1^ corresponded to the N–H stretching vibration. The band found at 1472 cm^−1^ was characteristic of N–H deformation vibration. The C–N stretching had bands are at ~ 1169, 1087, and 1043 cm^−1^. The (CH_3_)_2_N absorption presented a band at ~ 2821 cm^−1^. Other bands at 744 and 634 cm^−1^ corresponded to NH_2_ vibration and CH bending, respectively (50,51).

**Fig. 10:**
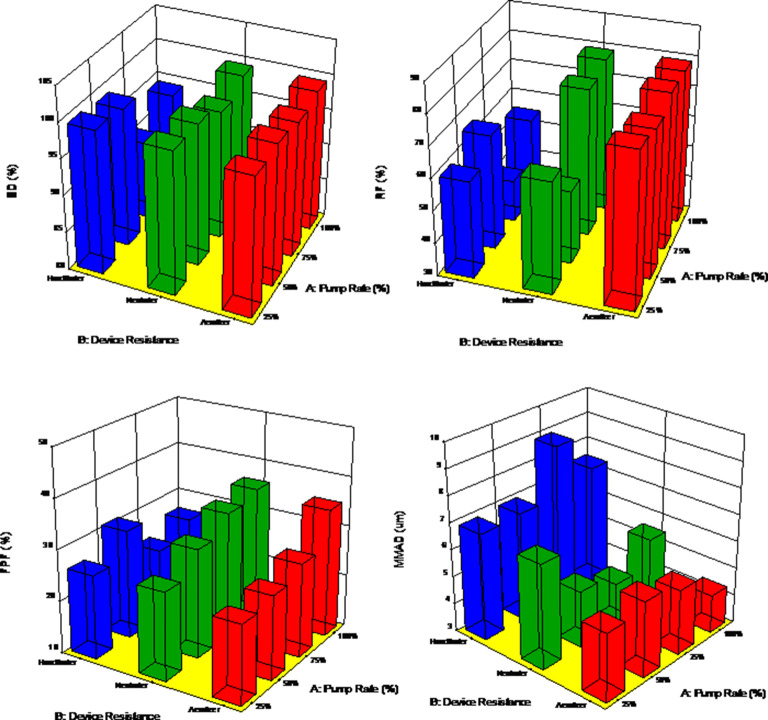
3D surface response plots by Design-Expert® 8.0.7.1 software (Stat-Ease Corporation, Minneapolis, MN, USA) displaying the influence and interplay of pump rate and different DPI devices on *in vitro* aerosol dispersion parameters for SD metformin dry powder formulations for **a** ED, **b **RF, **c **FPF, and **d **MMAD

The *in vitro* aerosol dispersion performance (Fig. 5) and statistical analyses gave interesting and promising results. All of the aerosol dispersion parameters (Supplementary Material Table 7) evaluated in the SD powders with the three devices had values much higher than those already on the market. The ED was almost 99% for all formulations meaning that the design of a DPI device (the flow path of the device between the loaded powder and the exit of the mouthpiece of the device) was efficient with the three different tested devices allowing consistent and sufficient release of the amount of powder at the tested flow rate (35). The deposition in stages 2–7 was measurable and reflected in the FPF. These values were much higher than “conventional” DPIs currently on the market, which provide an FPF in the range of 10–20%. The MMAD values for all formulations using the three devices were optimal for DPIs with formulations having calculated MMAD values ≤ 5 μm which makes them suitable for deposition into the deep airways. Furthermore, the statistical analysis and the 3D plots showed only minimal differences between the performance using different DPI devices and different SD pump rates. Statistical analysis of the interaction between the formulations made by advanced spray drying parameters and the type of DPI device was also performed. The 3D surface plots shown in Fig. 10 illustrate these interactions and the interplay between the various parameters. The ANOVA for the ED values indicated that there was no statistically significant difference between the different PRs and the three different DPI devices. However, for RF values, there was a statistically significant difference between the SD metformin PR formulations and the different DPI devices (*p* value < 0.0001), with the Aerolizer® (low-medium shear stress device) giving the highest RF values. For FPF values, there was also a statistically significant difference (*p* value = 0.0252) using different devices and different SD metformin PR formulations, with the Neohaler™ and the Aerolizer® giving the highest FPF for the SD metformin 100% PR formulation. The MMAD calculated aerodynamic property was a statistically significant difference with a *p* value = 0.0001, for lowest MMAD, it is achieved with the Aerolizer® DPI device.

Taking the differences in the resistance and the geometry of the three different tested devices, we conclude that devices with low and medium resistance are better for these metformin formulations. This could be due to the particles having optimal physicochemical properties and not needing a very high resistance device to fluidize the particles and aerosolize them. Finally, our data obtained from *in vitro* cell assays demonstrated that the SD formulations of metformin are safe and do not damage the integrity of the epithelium of the tested 2D and 3D pulmonary cell lines even when exposed to high concentrations of Metformin.

## CONCLUSIONS

We report, for the first time, a systematic study demonstrating that inhalable solid-state nanoparticles/microparticles of metformin, an AMPK and Nrf2 activator, can be successfully designed and produced using advanced spray drying conditions in closed mode. In addition, we were able to demonstrate that these inhalable solid-state nanoparticles/microparticles have the essential particle properties needed for delivery as DPIs with efficient aerosolization and high aerosol dispersion performance. Metformin was shown to retain crystallinity following advanced spray drying under these conditions. When integrated with three FDA-approved human DPI devices with varying device shear stress, all nanoparticle/microparticle powders were successfully aerosolized with high aerosol dispersion and resulted in deposition on the lower NGI stages indicating the very small aerodynamic size range which is necessary for efficiently targeting the small airways and deep lung region. The interplay between spray drying pump rate properties, particle properties, DPI device shear stress properties, and aerosol dispersion properties was demonstrated in a meaningful manner. In addition, safety function of the dose was successfully demonstrated in various human cell lines both as 2D cell culture and 3D human small airway epithelia composed of primary cells at the ALI. Thus, we conclude that inhalable nanoparticles/microparticles of metformin could be developed as therapies to treat complex pulmonary diseases that currently have sub-optimal therapeutic options.

## Supplementary Information

ESM 1 (DOCX 525 kb)
