# Supplementary material for: Inhalable Nanoparticles/Microparticles of an AMPK and Nrf2 Activator for Targeted Pulmonary Drug Delivery as Dry Powder Inhalers
Source: AAPS J. 2020 Nov 16;23(1):2. doi: 10.1208/s12248-020-00531-3 (PMC7669792; doi:10.1208/s12248-020-00531-3)
Supplement: Supplementary file 1 — (DOCX 525 kb) [file 12248_2020_531_MOESM1_ESM.docx]

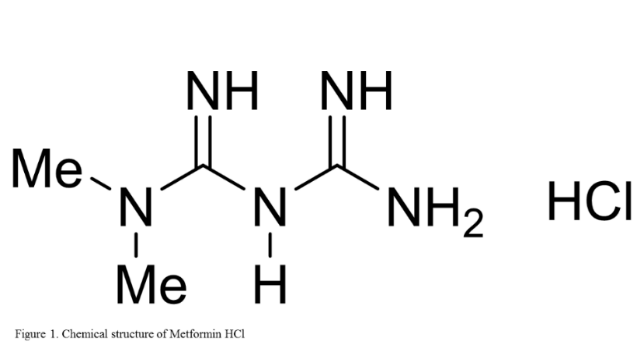


**Supplementary Material Figure 1.** Chemical structure of Metformin HCl (ChemDraw™ Ultra Ver. 15.0.; CambridgeSoft, Cambridge, MA, USA)


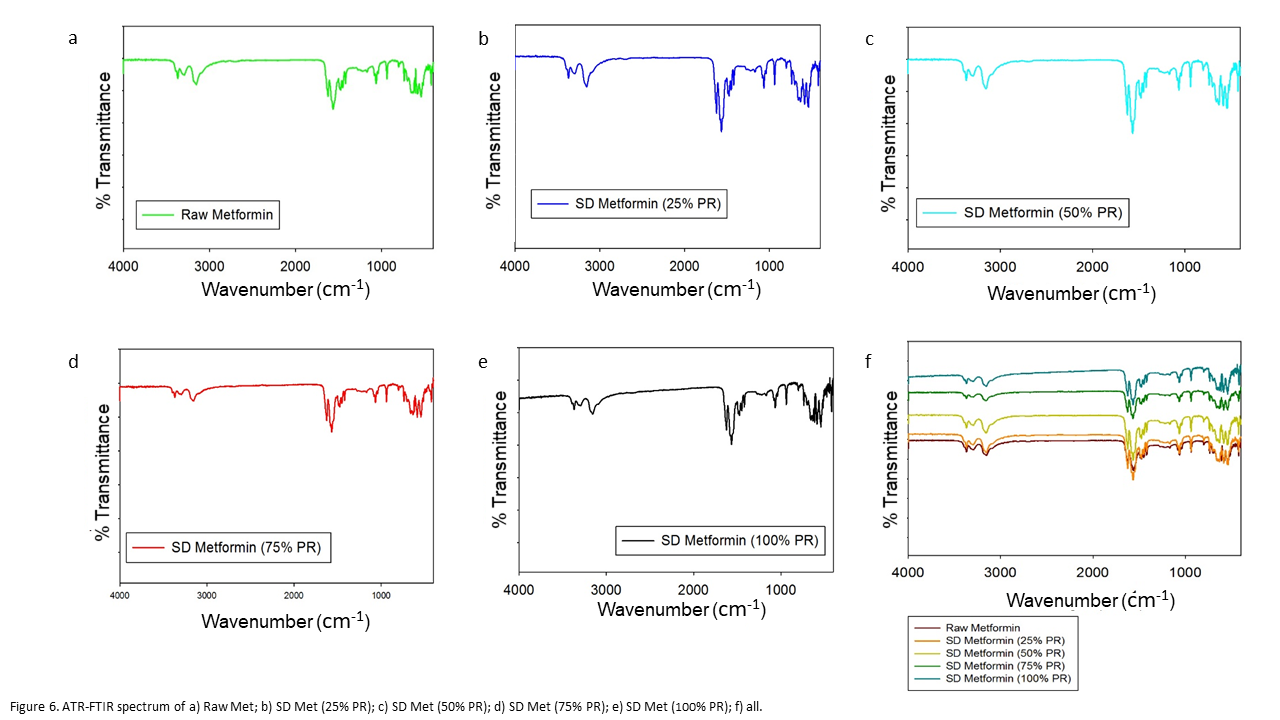


**Supplementary Material Figure 2.** ATR-FTIR spectra of : a) Raw Metformin HCl; b) SD Met (25% PR); c) SD Metformin (50% PR); d) SD Metformin (75% PR); e) SD Metformin (100% PR); and f) all.

**
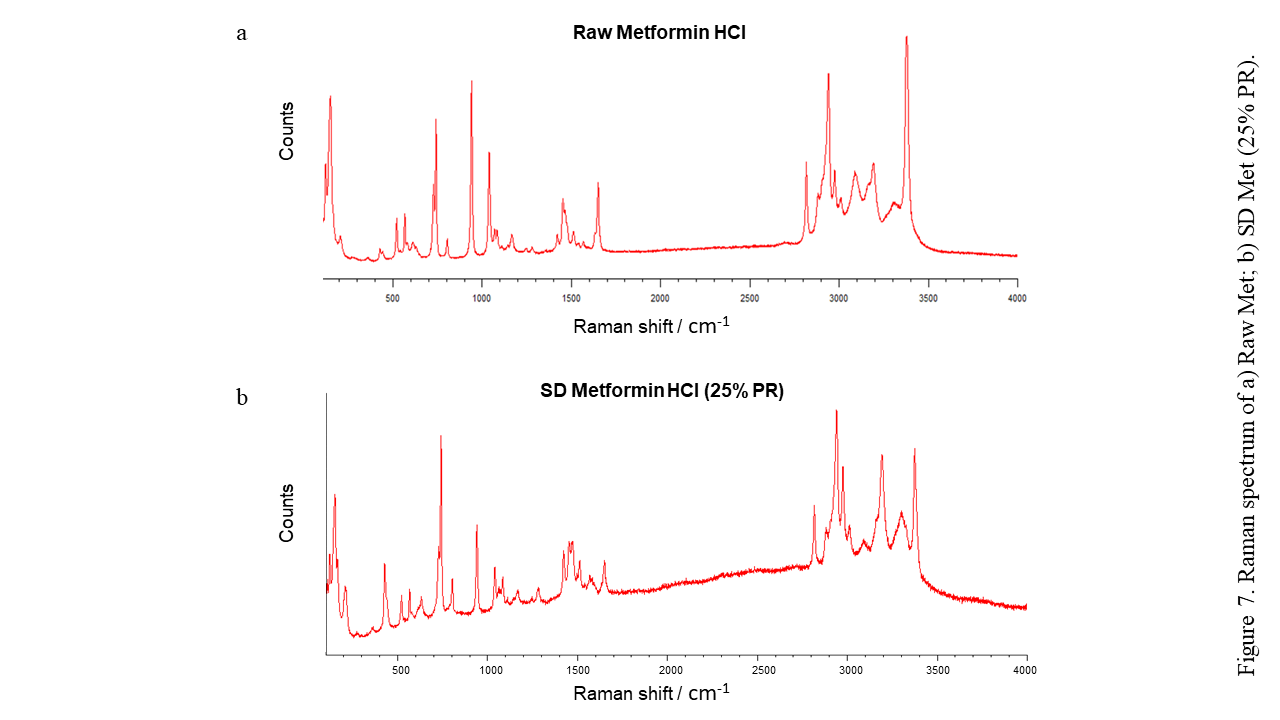
**

**Supplementary Material Figure 3.** Raman spectra of: a) Raw Metform HCl and b) SD Metformin (25% PR).

| **Spray Drying Conditions** | |
| --- | --- |
| **Parameter** |  |
| **Inlet Temperature** | 150 ˚C |
| **Aspirator Rate** | 100%  40 m^3^/hour |
| **Pump rate % (Feed Rate)** | 25% (7.5 mL/min)  50% (15 mL/min)  75% (22.5 mL/min)  100% (30 mL/min) |
| **Gas Flow** | 670 L/hour  55 mm Hg |
| **Feed Solution Concentration** | 0.1% w/v |
| **Solvent** | Methanol |
| **Atomizer and Drying gas** | UHP Nitrogen |
| **Nozzle type and diameter** | Stainless steel 0.7mm |

**Supplementary Material Table I.** Advanced spray drying parameters for metformin spray dried (SD) powders from methanol (MeOH) solution using advanced organic solution closed-mode spray drying particle engineering design.

**Supplementary Material Table II.** Spraying drying pump rates with corresponding outlet temperatures.

| **System Composition** | **Outlet Temperature (°C)** |
| --- | --- |
| SD Metformin (25% PR) | 80-83 |
| SD Metformin (50% PR) | 64-65 |
| SD Metformin (75% PR) | 62 |
| SD Metformin (100% PR) | 53 |

**Supplementary Material Table III.** Particle size volumetric diameter (D_v_) by laser sizer diffraction for SD metformin particles (mean ± SD, n=3).

| **System Compositon** | **D_v10_ (µm)** | **D_v50_ (µm)** | **D_v90_ (µm)** | **Mean ± std dev (µm)** | **Span** |
| --- | --- | --- | --- | --- | --- |
| SD Metformin (25% PR) | 0.266±0.05 | 0.348±0.05 | 0.522±0.080 | 0.363±0.059 | 0.735 |
| SD Metformin (50% PR) | 0.652±0.09 | 0.897±0.14 | 1.366±0.22 | 0.959±0.16 | 0.795 |
| SD Metformin (75% PR) | 0.357±0.05 | 0.46±0.08 | 0.685±0.12 | 0.482±0.08 | 0.712 |
| SD Metformin (100% PR) | 0.469±0.12 | 0.621±0.18 | 0.937±0.28 | 0.653±0.19 | 0.752 |

**Supplementary Material Table IV.** Particle size diameters by image analysis. (n=100, Mean ± SD).

| **System Composition** | **Mean Size (µm) ± Std Dev** | **Range (µm)** |
| --- | --- | --- |
| Raw Metformin HCl | 281.49±135.35 | 797.85-94.32 |
| SD Metformin (25% PR) | 5.622±2.397 | 0.859-17.547 |
| SD Metformin (50% PR) | 1.1±0.295 | 0.552-1.805 |
| SD Metformin (75% PR) | 3.134±1.503 | 0.888-8.465 |
| SD Metformin (100% PR) | 3.147±2.279 | 0.721-12.285 |

**Supplementary Material Table V.** DSC phase transition values for raw and SD metformin powders. (n = 3, Mean ± SD).

| **System Composition** | **T_peak_ (⁰C)** | **Enthalpy (J/g)** |
| --- | --- | --- |
| Raw Metformin HCl | 226.81±0.16 | 312.2±5.20 |
| SD Metformin (25% PR) | 225.29±0.01 | 306.9±7.45 |
| SD Metformin (50% PR) | 225.54±0.35 | 300.43±11.94 |
| SD Metformin (75% PR) | 225.64±0.42 | 301.6±6.58 |
| SD Metformin (100% PR) | 225.58±0.42 | 302.6±6.58 |

**Supplementary Material Table VI.** KFT residual water content values (n=3, mean ± SD) for SD metformin powders.

| **System Composition** | **Residual Water content (% w/w)** |
| --- | --- |
| Raw Metformin HCl | 0.33±0.13 |
| SD Metformin (25% PR) | 3.41±2.39 |
| SD Metformin (50% PR) | 1.31±1.28 |
| SD Metformin (75% PR) | 1.74±0.03 |
| SD Metformin (100% PR) | 1.45±0.34 |

**Supplementary Material Table VII.** *In vitro* aerosol dispersion parameters including mass median aerodynamic diameter (MMAD), geometric standard deviation (GSD), fine particle fraction (FPF), respirable fraction (RF), and emitted dose (ED). (n=3, *mean ± SD).*

| **System Composition** | **ED**  **(%)** | **FPF**  **(%)** | **RF**  **(%)** | **MMAD**  **(μm)** | **GSD**  **(μm)** |
| --- | --- | --- | --- | --- | --- |
| **Aerolizer^®^** | | | | | |
| SD Metformin (25% PR) | 99.11±19 | 25.42±2.11 | 79.38±1.31 | 5.57±0.17 | 1.90±0.05 |
| SD Metformin (50% PR) | 99.56±0.31 | 26.58±1.19 | 77.18±2.17 | 5.82±0.12 | 1.81±0.07 |
| SD Metformin (75% PR) | 98.88±1.04 | 28.66±2.08 | 80.29±0.89 | 5.4±0.19 | 1.84±0.04 |
| SD Metformin (100% PR) | 99.47±0.23 | 52.06±3.89 | 92.09±0.27 | 3.61±0.51 | 1.79±0.07 |
| **NeoHaler^™^** | | | | | |
| SD Metformin (25% PR) | 99.72±0.49 | 27.39±1.44 | 65.57±5.5 | 6.9±0.36 | 1.78±0.08 |
| SD Metformin (50% PR) | 99.87±0.22 | 32.05±2.46 | 53.56±1.36 | 5.12±0.29 | 2.25±0.04 |
| SD Metformin (75% PR) | 97.91±1.80 | 35.08±4.73 | 77.48±6.23 | 4.62±1.24 | 1.87±0.1 |
| SD Metformin (100% PR) | 99.87±0.22 | 26.64±0.97 | 61±2.17 | 5.60±0.57 | 1.79±0.07 |
| **HandiHaler^®^** | | | | | |
| SD Metformin (25% PR) | 99.87±0.22 | 26.64±0.67 | 61±2.17 | 7±0.32 | 1.91±0.8 |
| SD Metformin (50% PR) | 99.31±0.63 | 31.68±3.5 | 66.87±4.05 | 7.07±0.58 | 2.40±0.70 |
| SD Metformin (75% PR) | 91±7.97 | 24.03±8.1 | 43.53±13.16 | 8.95±1.30 | 2.47±0.22 |
| SD Metformin (100% PR) | 94.74±9.10 | 27.36±6.05 | 55.95±4.12 | 7.53±0.16 | 1.86±0.11 |
